# Uncertain future for global sea turtle populations in face of sea level rise

**DOI:** 10.1038/s41598-023-31467-1

**Published:** 2023-04-20

**Authors:** Marga L. Rivas, Emilio Rodríguez-Caballero, Nicole Esteban, Antonio J. Carpio, Barbara Barrera-Vilarmau, Mariana M. P. B. Fuentes, Katharine Robertson, Julia Azanza, Yolanda León, Zaida Ortega

**Affiliations:** 1 Biology Department, Marine Research Institute INMAR, University of Cádiz, Cádiz, Spain; 2 Agronomy Department of the University of Almería and Research Centre for Scientific Collections from the University of Almería (CECOUAL), Almería, Spain; 3 Bioscience Department, Swansea University, Wales, SA2 8PP UK; 4 SaBio Research Group, Instituto de Investigación en Recursos Cinegéticos (IREC-CSIC-UCLM-JCCM), Ciudad Real, Spain; 5 Mondonguillo-Laguna Urpiano NGO, Matina, Costa Rica; 6 Marine Turtle Research, Ecology and Conservation Group, Department of Earth Ocean and Atmospheric Science, Florida State University, Tallahassee, FL USA; 7 Department of Environment and Science, Queensland Government, Brisbane, Australia; 8 University of Habana, Habana, Cuba; 9 Technological Institute of Santo Domingo INTEC, Santo Domingo, Dominican Republic; 10 Department of Ecology and Conservation, Federal University of Mato Grosso do Sul, Campo Grande, Brazil; 11 Department of Zoology, University of Granada, Granada, Spain

**Keywords:** Climate sciences, Ecology, Conservation biology, Ecosystem ecology

## Abstract

Sea level rise has accelerated during recent decades, exceeding rates recorded during the previous two millennia, and as a result many coastal habitats and species around the globe are being impacted. This situation is expected to worsen due to anthropogenically induced climate change. However, the magnitude and relevance of expected increase in sea level rise (SLR) is uncertain for marine and terrestrial species that are reliant on coastal habitat for foraging, resting or breeding. To address this, we showcase the use of a low-cost approach to assess the impacts of SLR on sea turtles under various Intergovernmental Panel on Climate Change (IPCC) SLR scenarios on different sea turtle nesting rookeries worldwide. The study considers seven sea turtle rookeries with five nesting species, categorized from vulnerable to critically endangered including leatherback turtles (*Dermochelys coriacea*), loggerhead turtles (*Caretta caretta*), hawksbill turtles (*Eretmochelys imbricata*), olive ridley turtles (*Lepidochelys olivacea*) and green turtles (*Chelonia mydas*). Our approach combines freely available digital elevation models for continental and remote island beaches across different ocean basins with projections of field data and SLR. Our case study focuses on five of the seven living sea turtle species. Under moderate climate change scenarios, by 2050 it is predicted that at some sea turtle nesting habitats 100% will be flooded, and under an extreme scenario many sea turtle rookeries could vanish. Overall, nesting beaches with low slope and those species nesting at open beaches such as leatherback and loggerheads sea turtles might be the most vulnerable by future SLR scenarios.

## Introduction

Climate change has accelerated sea level rise (SLR) since the 1970s and is now more rapid than the mean SLR rate recorded during the previous two millennia^1–3^. By the end of this century it is projected that SLR will reach 82 cm^2^ and—in extreme scenarios—could exceed 2 m^3^ with the early onset of Antarctic ice sheet instability^4^. Regional variation in predictions in SLR show that tropical regions and small islands are among the most vulnerable^5^, threatening species that depend on these coastal habitats, such as sea turtles^6–8^. Sea turtle species exhibit natal philopatry, returning to the beach where they were born^9^ with exceptionally high precision for returns to island rookeries^10^. However, climate changes might be too rapid for sea turtles to respond through their ability to disperse or colonize new habitats^11^. These biological traits and their reliance on sandy beaches make them particularly vulnerable to changes in coastal areas, like those resulting from SLR.

As a result, concern exists on the potential impacts of SLR on sea turtles, however only a dozen studies to date have projected how SLR will impact them^12^. These previous studies have been mainly regionally-focused, including assessments from only one or two species^7,13–16^. This regional focus is likely a result from the challenges inherent in successfully assessing shoreline response to SLR^17,18^. Although most sea turtle assessments have been obtained from field survey methods, such studies of estimations of stream reach water surface slopes often have low accuracy^19^. Other approaches which couple LiDAR with biological data^20,21^ have higher accuracy, but are also more costly^22^. However, new methodologies, such as the use of open Digital Elevation Models (DEMs) might be a good proxy broadly applicable to assess SLR by satellite images^23^.

**Figure 1:**
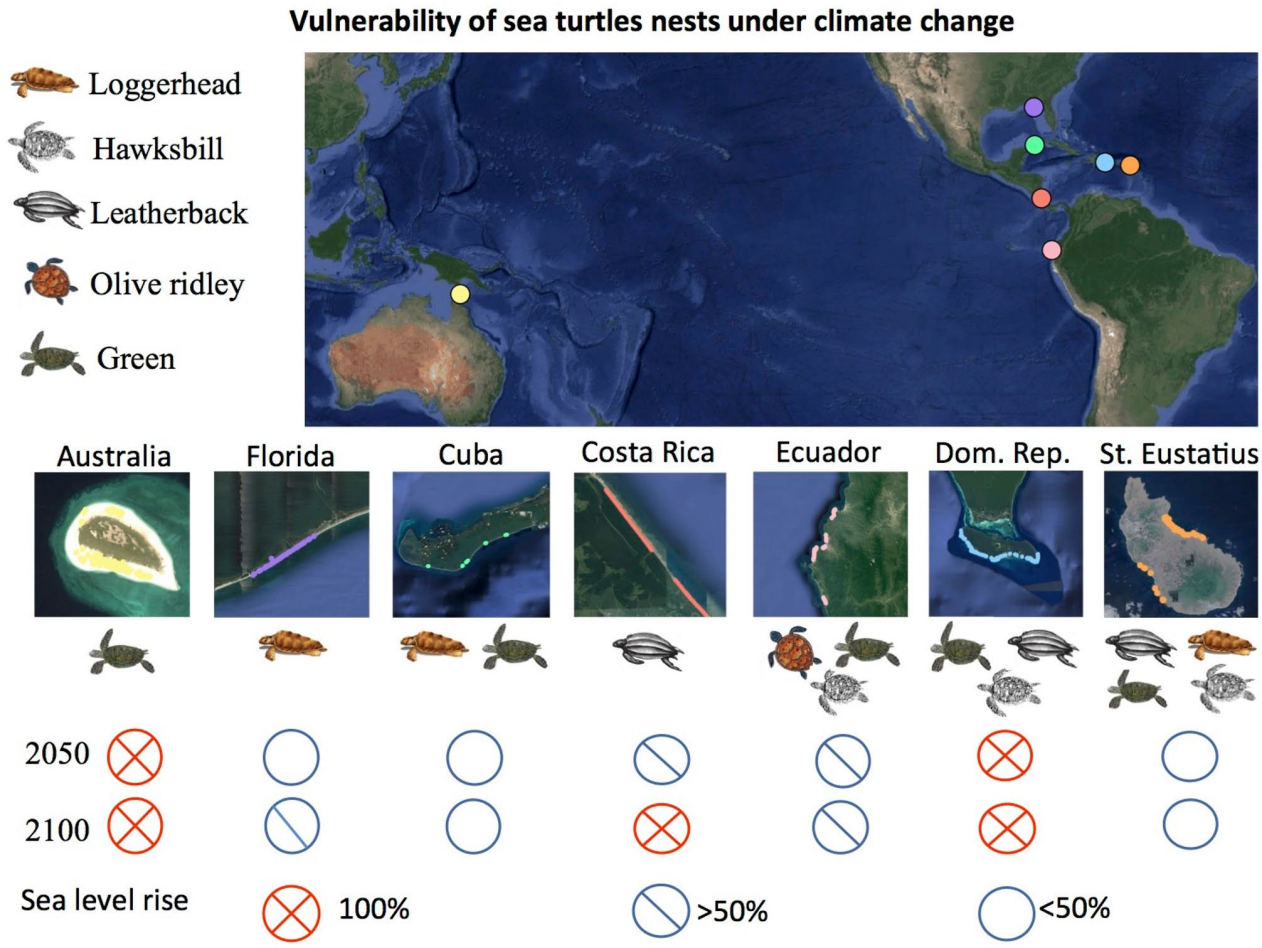
Vulnerability of sea turtle nests under sea level rise at IPCC’s RCP 4.5. Seven study sites at sea turtle rookeries spanning countries in the Caribbean Sea, Gulf of Mexico and Pacific Ocean, with five species represented. In the Caribbean and Gulf of Mexico: Mondonguillo beach, Costa Rica; Guanahacabibes peninsula, Cuba; Saona Island, Dominican Republic; Zeelandia, Turtle, Kay bay, Tumbledowndick, Crooks and Oranjebaai beaches, St Eustatius; and St George Island, Florida, USA. In the Pacific Ocean: Coast of Ecuador; and Raine Island, Australia. Map Data: Google Earth and free images.

Considering that most sea turtle rookeries across the globe are located in remote areas in low and middle-income countries, less costly approaches for field surveys are often preferred and can provide baseline data to identify areas at most risk. Indeed, the few studies assessing the impacts of SLR on sea turtles to date discuss the challenges inherent in successfully predicting shoreline response to SLR and storm activities^17,18^ and the inability to couple projections with biological information such as sex ratios and reproductive success^12^. Here we present an assessment of the potential impact of SLR on sea turtle rookeries by applying a low-cost methodology to estimate the probability of flooding of nest locations under multiple IPCC SLR scenarios. This approach combines turtle nest locations, freely available DEMs and Climate Central maps under Coastal DEM predictions^22^. The study considers seven sea turtle rookeries with five nesting species, categorized from vulnerable to critically endangered^24^ including leatherback turtles (*Dermochelys coriacea*), loggerhead turtles (*Caretta caretta*), hawksbill turtles (*Eretmochelys imbricata*), olive ridley turtles (*Lepidochelys olivacea*) and green turtles (*Chelonia mydas*) (Fig. 1; Extended Data Table 1). Our study sites encompass some important nesting sites for sea turtles globally (e.g., Raine Island, Australia, the largest green turtle rookery worldwide^25^) and have different characteristics (i.e., beach width, slope, size), which will allow us to estimate SLR effects on a wide range of nesting rookeries and highlights the broad applicability of our approach.

## Vulnerability of sea turtle nests to SLR

**Figure 2:**
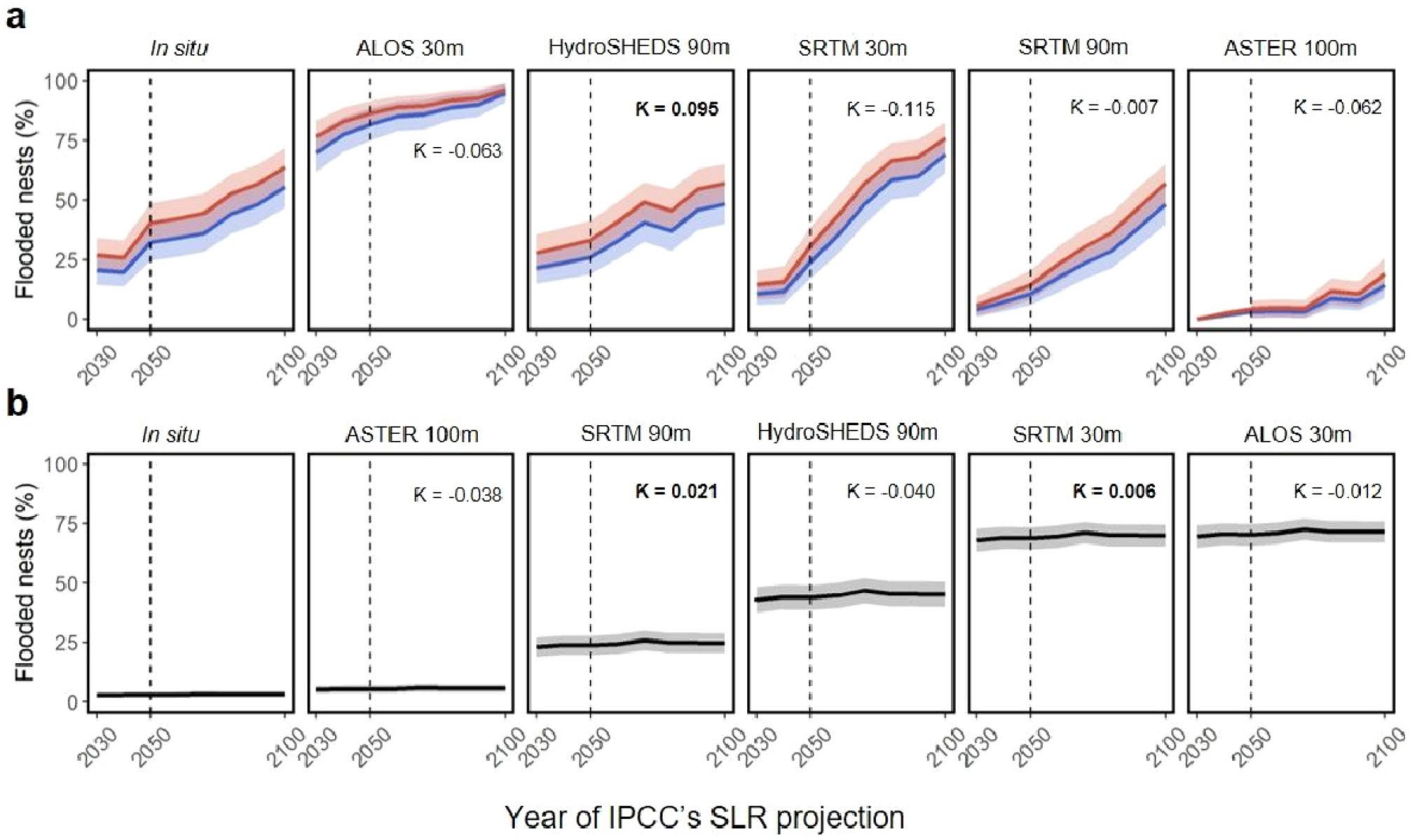
Summary of model predictions. Predictions of the models for different DEMs and empirical data (in situ) indicate the best fit of probability of nest flooding for (**a**) Costa Rica and (**b**) Ecuador turtle nests. IPCC sea level rise predictions are included for RCP 4.5 (blue) and RCP 8.5 (red). For Ecuador, the RCP climate change scenario did not influence the probability of nest flooding. Cohen’s kappa (κ) values for prediction (or not) of flooding for each nest compared in situ data with respective DEM datasets. Bold values correspond to a small but significant relationship between the in situ and DEM data.

**Figure 3:**
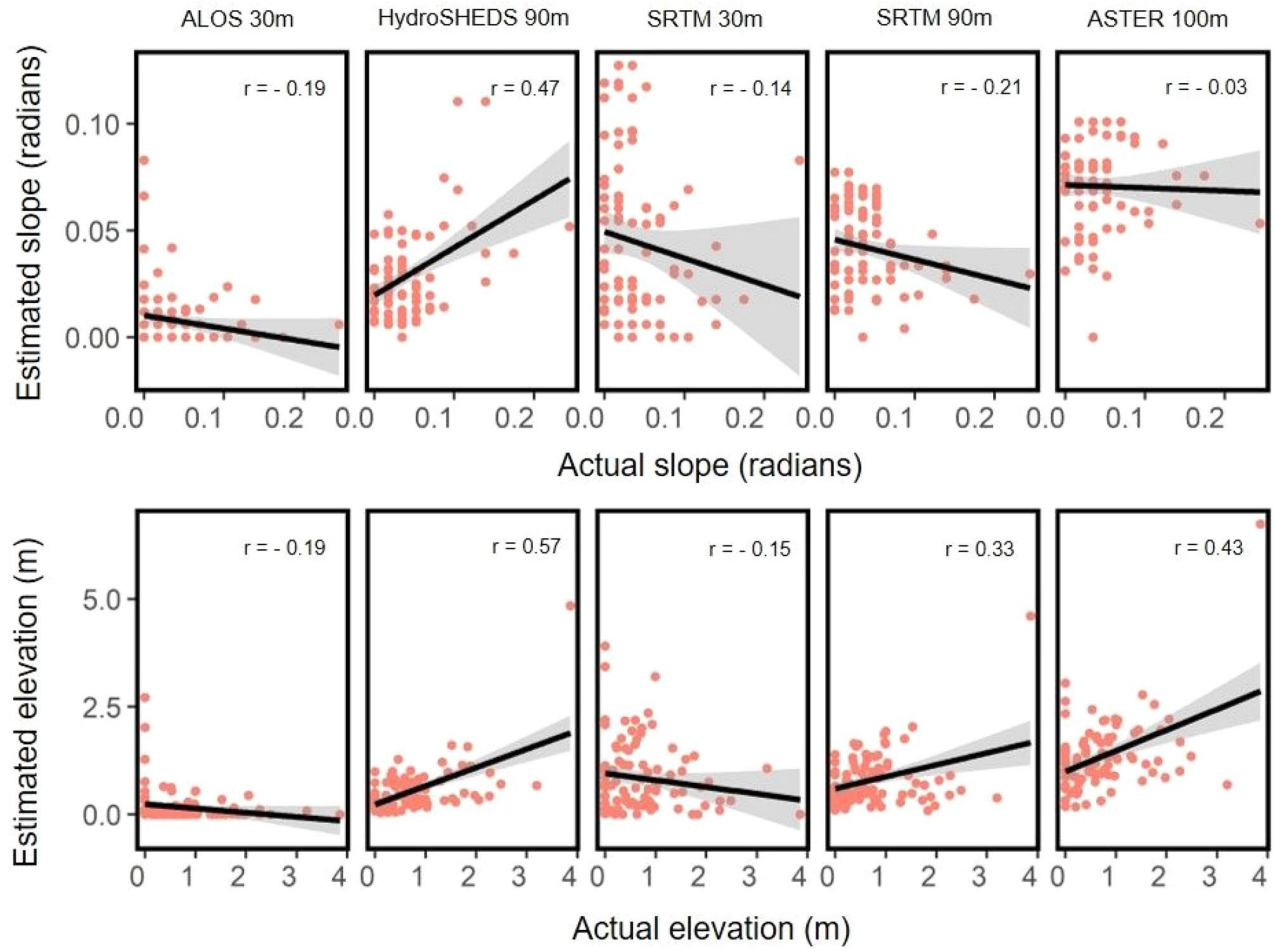
Summary of actual vs. estimates of slope and elevation by DEM. Comparison of the relationship between slope (radians) and elevation (m) using empirical data (in situ measurements) and DEMs for turtle nesting beaches in Costa Rica.

From GPS locations of 2835 marine turtle nests belonging to five different species from seven study areas across the globe, we estimated the vulnerability of nests to flooding considering available projections of SLR caused by climate change from 2010 to 2100 (Fig. 1). Firstly, we used data from GPS nest locations + available DEMs + IPCC projections for the seven studied populations across the globe to assess and compare the suitability of the different DEMs to make flooding predictions. The comparison of freely available elevation data shows that the mean estimated elevation for nest sites (and consequently mean proportion of flooded nests) differed substantially between DEM sources for each rookery (Extended Data Fig. 2). It was expected that the highest resolution (30 m) DEMs would be most accurate (compared with lower resolutions of 90–100 m) but there were similarities in accuracy amongst DEMs for each study site (Fig. 2; Extended Data Figs. 3 and 4). In addition, Cohen’s kappa results showed weak relationships between DEM model predictions and the in situ data (Fig. 3; Supplementary Table 3).

**Figure 4:**
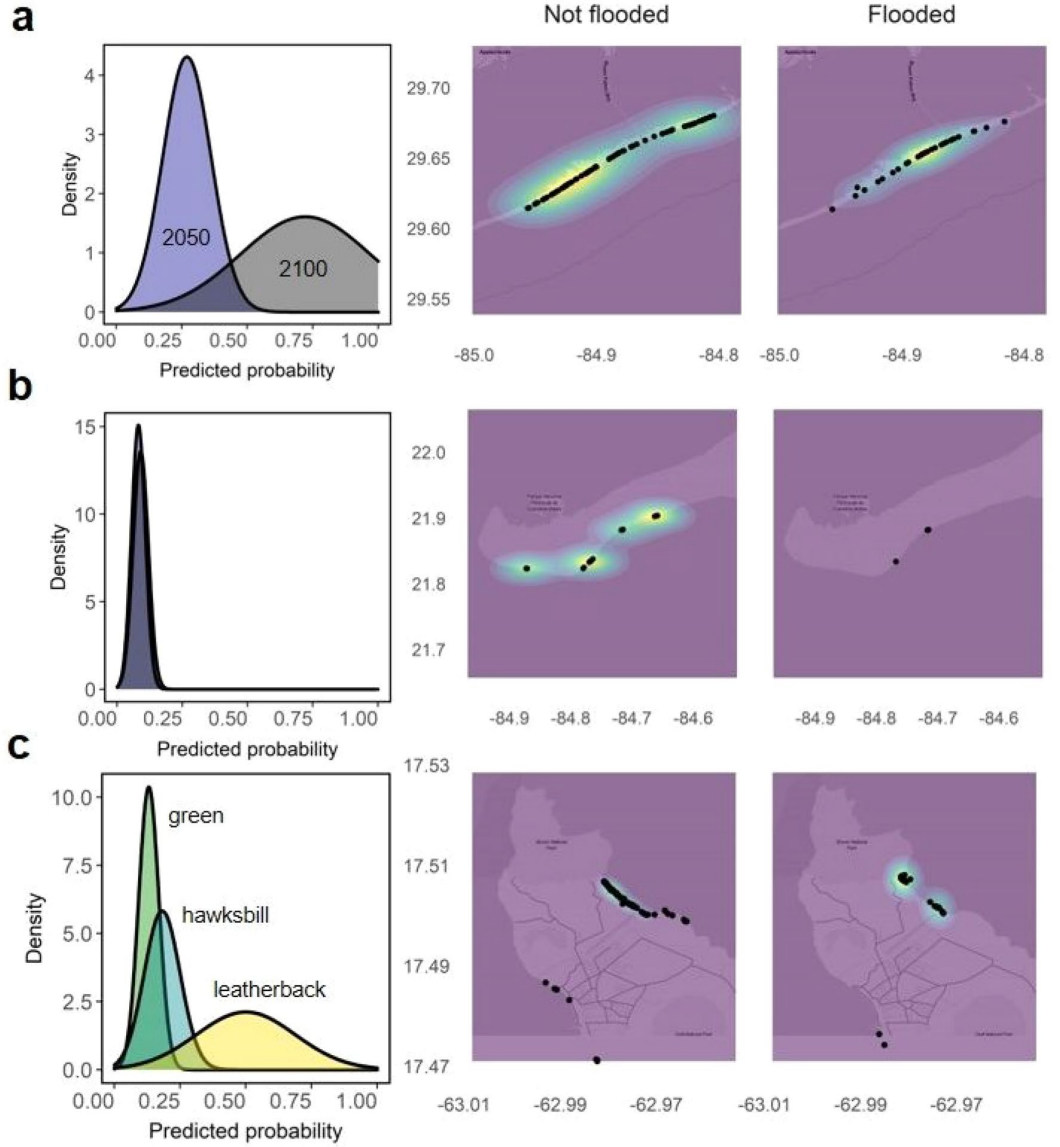
Predictions of sea turtle nest flooding in a subset of rookeries. Sites include (**a**) Florida (USA) (2050 and 2100), (**b**) Cuba (2050), and (**c**) St Eustatius (three species, 2050). Column 1: Proportion of nests likely to be flooded at each location. Column 2: Heatmap of nests predicted to be free from flooding by 2050. Column 3: Heatmap of nests predicted to be flooded by 2050, identifying the areas at highest risk. Probability values of 1 (yellow), 0.5 (blue) and 0 (purple) represent the density of unflooded or flooded nests within respective heatmaps. A Climate Central moderate scenario was adopted for these scenarios (Kopp et al. 2017).

Then, we estimated the probability of flooding from GPS nest locations georeferenced on CoastalDEM maps (e.g., Extended Data Fig. 5) for five of the seven study areas, since CoastalDEM did not provide reliable estimates in Costa Rica and Ecuador (e.g., Extended Data Fig. 6). For the other five study areas, predictions from Coastal DEM maps (Fig. 4) and from basic in situ data (Supplementary Tables 8–15) were reliable to predicting nest flooding under SLR. Raine Island (Australia) and Saona Island (Dominican Republic) are the most vulnerable populations with 100% nest flooding predicted under moderate emissions scenarios for 2050 (Fig. 1; Supplementary Table 4). For Florida, flooding probability greatly increases after 2050 (Fig. 2a; Supplementary Table 5). Cuba, probably thanks to their elevated beaches, showed the lowest vulnerability to flooding throughout the twenty-first century (Fig. 2b; Supplementary Table 6). For St. Eustatius, main differences in flooding vulnerability arise from differences between turtle species (see Fig. 4c and next section).

Overall, our results suggested that flat islands and cays may be highly vulnerable to sea level rise under the moderate IPCC scenario (Fig. 1; Supplementary Table 4)^7,26^ predominantly those in the Caribbean and Pacific^27,28^. Some of these locations are important rookeries^25,29^ for species which return every 1–4 years to nest at the beach where they were born^9^. Furthermore, some of them host nestings for more than one species and present several nesting environments and beach characteristics such as different slopes, width, or sand grain size, among others^30–32^.

A number of rookeries subjected to beach erosion have already been assessed as vulnerable due to loss of beaches used for nesting^8,33^, nest loss^21,32^ and changes in nesting behaviour^16^. The philopatry of leatherbacks^34^ and loggerheads is not quite strict^35^ and they can move great distances and nest further up the beach in response to SLR depending on future beach availability. However, it has already been reported that 20% of Costa Rican leatherbacks nest in flooded areas when scarp barriers were present^30^. The expected habitat loss rates found in the study areas could have important effects on nesting success since philopatry could lead many individuals to nest on inundated beaches. An unknown variable is the potential for sea turtles to adapt to new scenarios. Therefore, their survivorship will depend on their resilience and adaptability to rapid changes within their nesting habitats^36^.

## Vulnerability of nests across species

To determine differences between modelled nest flooding probabilities by species we assessed rookeries containing multiple species (i.e., St Eustatius, Dominican Republic and Ecuador rookeries; Fig. 1). Overall, we identified that leatherback turtle nests may be at a higher risk from flooding compared to the rest of the studied species because they tend to nest in open areas near to the high tide line (Fig. 4c). In St Eustatius, significant variation exists between nest flooding probability by species (Supplementary Table 7): leatherback turtle nests are at the highest risk from flooding compared with green turtles (ß = 1.892, p < 0.001; Fig. 4c), and there was no significant difference between flooding risk for hawksbill and green turtle nests (ß = 0.388, p = 0.339; Supplementary Table 3). The model predicted that, on average, 50.0% leatherback, 18.2% hawksbill and 13.1% of green turtle nest locations would be flooded by 2050 (Fig. 1; Supplementary Table 4). The predicted model accuracy for estimation of likelihood of nest flooding was 79.4%. Leatherback turtle nests in open areas of beaches have been found to already been subject to occasional flooding^30^, whilst hawksbill and green turtles tend to nest at higher elevations closer to dunes and steep cliffs along the coastline^37^ and olive ridleys nest at open beaches with low slopes^38^. This difference in nesting location might explain those differences in nest location flooding potential between species. In consequence, leatherback turtle populations as well as other turtles nesting at open beaches (e.g., loggerhead turtles) may be at greater risk from SLR than other species.

In Ecuador, no differences in modelled nest flooding severity were found between olive ridley, greens and hawksbill species, potentially due to beaches at this location being steeper^32^ (Supplementary Table 15). Similarly, no difference in nest flooding was predicted between nests of different species in the Dominican Republic, although only because all locations were estimated to be inundated by 2050 (Fig. 1; Supplementary Table 3; Supplementary Table 4). It is likely that the potential effect of SLR will vary between species, potentially linked to variables such as nesting beach characteristics (e.g., slope, aspect^39^), nesting habitat preferences and suitable nesting areas^33^.

## Conclusions—conservation concerns

Our models of nest flooding validated by field data considered that even a moderate increase in greenhouse emissions (RCP 4.5)^3^ might impact the reproductive output of sea turtles at the rookeries included in our study. Recent predictions of accelerating global SLR due to rapid melting of ice in Greenland^40^ and the Antarctic^41^ in combination with ocean currents^42^ indicate that pessimistic scenarios could be more accurate than conservative scenarios^43^. Such scenarios support our projections by indicating that sea turtle nesting populations could be vulnerable to flooding under even moderate scenarios over the next decades.

Relatively recent methods of remote sensing and modeling including DEMs^4,43,44^, drones, photogrammetry and GPS have been adopted to assess impacts of SLR on sea turtle populations^12^. However, most highly accurate methodologies entail high costs (e.g., 1500–15,000€ per satellite image) limiting their use to more localised studies. Considering that most sea turtle nesting populations around the world are located in low and middle-income countries, local conservation projects cannot afford the costs of these intensive methodologies to assess the vulnerability of nesting beaches. We have demonstrated that a methodology based on low-cost technological models can be a useful tool for predicting possible future SLR scenarios in important sea turtle nesting areas. We highlighted the utility of global open DEM data with high accuracies for remote areas that could assist with estimation of the vulnerability of sea turtle nesting populations worldwide.

Scientific assessments are essential for prediction of the impacts of future climate scenarios and to assist stakeholders and managers in anticipating extreme scenarios of coastal erosion or flooding, and to predict areas at higher risk of flooding^45^. Such assessments will help identify conservation refugia and nesting beaches that have greater resilience to climate change^36^. Although sea turtles have been around for millions of years and would be present in several climate change events, we do not know how their populations might be affected by these projected rapid changes of high loss of nesting sites in the study areas by 2050. Thus, this demonstrates the urgency of developing a multi-species assessment at a global scale in order to develop conservation plans for the most vulnerable populations while there is still time. Conservation management strategies are already in place to enhance resilience to SLR at some nesting beaches, including sand refilling of nesting beaches^46^ such as in Raine Island, relocation of nests to safe places^47^ or the protection of hatcheries for rookeries with extreme erosion and flooding^31^. In addition, we highlighted the need for climate change adaptation measurements to be implemented in management plans considering estimated projections under moderate SLR scenarios.

However, if the world maintains current carbon dioxide emission rates, worst-case scenarios might be vastly underestimated by 3–4 times^48^ and existing management strategies may then be insufficient to protect the future of many sea turtle populations worldwide. In summary, our study predicts massive flooding at important rookeries in Australia, Dominican Republic, Costa Rica and the USA. These critical areas will face the effects of SLR in the next few decades, meaning that it is now urgent to reduce anthropogenic emissions to safeguard the future of sea turtle populations against climate change and associated sea level rise.

## Supplementary Information

Supplementary Information.

## Data Availability

The datasets used and/or analysed during the current study available from the corresponding author on reasonable request.

## References

[1] Frederikse T, Buchanan MK, Lambert E, Kopp RE, Oppenheimer M, Rasmussen DJ, van de Wal RS (2020). Antarctic Ice Sheet and emission scenario controls on 21st-century extreme sea-level changes. Nat. Commun..

[2] Intergovernmental Panel on Climate Change (IPCC). In *Climate Change 2013: The Physical Science Basis. Contribution of Working Group I to the Fifth Assessment Report of the Intergovernmental Panel on Climate Change* (eds Stocker, T. F. *et al*.) (Cambridge University Press, 2013).

[3] Intergovernmental Panel on Climate Change (IPCC). *In Climate Change 2022: The Physical Basis, 6th Assessment Report *(2022)

[4] Kopp RE, DeConto RM, Bader DA, Hay CC, Horton RM, Kulp S, Strauss BH (2017). Evolving understanding of Antarctic ice-sheet physics and ambiguity in probabilistic sea-level projections. Earth's Future.

[5] Nurse, L. A. et al. in *Climate change 2014: Impacts, adaptation, and vulnerability. Part B: Regional aspects. Contribution of working group II to the fifth assessment report of the intergovernmental panel on climate change* (eds Barros, C. B. et al.) 1613–1654 (Cambridge University Press, 2014).

[6] Daniels RC, White TW, Chapman KK (1993). Sea-level rise: Destruction of threatened and endangered species habitat in South Carolina. Environ. Manage..

[7] Fuentes MMPB, Limpus CJ, Hamann M, Dawson J (2010). Potential impacts of projected sea-level rise on sea turtle rookeries. Aquat. Conserv..

[8] Katselidis KA, Schofield G, Stamou G, Dimopoulos P, Pantis JD (2014). Employing sea-level rise scenarios to strategically select sea turtle nesting habitat important for long-term management at a temperate breeding area. J. Exp. Mar. Biol. Ecol..

[9] Carr A, Stancyk S (1975). Observations on the ecology and survival outlook of the hawksbill turtle. Biol. Conserv..

[10] Levasseur KE, Stapleton SP, Fuller MC, Quattro JM (2019). Exceptionally high natal homing precision in hawksbill sea turtles to insular rookeries of the Caribbean. Mar. Ecol. Prog. Ser..

[11] Pike DA (2013). Forecasting range expansion into ecological traps: Climate-mediated shifts in sea turtle nesting beaches and human development. Glob. Change Biol..

[12] Patrício AR, Hawkes LA, Monsinjon JR, Godley BJ, Fuentes MM (2021). Climate change and marine turtles: Recent advances and future directions. Endanger. Species Res..

[13] Baker JD, Littnan CL, Johnston DW (2006). Potential effects of sea level rise on the terrestrial habitats of endangered and endemic megafauna in the Northwestern Hawaiian Islands. Endanger. Species Res..

[14] Mazaris AD, Matsinos G, Pantis JD (2009). Evaluating the impacts of coastal squeeze on sea turtle nesting. Ocean Coast. Manage..

[15] Reece JS, Passeri D, Ehrhart L, Hagen SC, Hays A, Long C, Wolf S (2013). Sea level rise, land use, and climate change influence the distribution of loggerhead turtle nests at the largest USA rookery (Melbourne Beach, Florida). Mar. Ecol. Progress Ser..

[16] Veelenturf CA, Sinclair EM, Paladino FV, Honarvar S (2020). Predicting the impacts of sea level rise in sea turtle nesting habitat on Bioko Island, Equatorial Guinea. PLoS ONE.

[17] Cooper JAG, Pilkey OH (2004). Sea-level rise and shoreline retreat: Time to abandon the Bruun Rule. Glob. Planet Change.

[18] Von Holle B, Irish JL, Spivy A, Weishampel JF, Meylan A, Godfrey MH, Taylor NR (2019). Effects of future sea level rise on coastal habitat. J. Wildl. Manage..

[19] Isaak DJ, Hubert WA, Krueger KL (1999). Accuracy and precision of stream reach water surface slopes estimated in the field and from maps. North Am. J. Fish. Manag..

[20] Long TM, Angelo J, Weishampel JF (2011). LiDAR-derived measures of hurricane- and restoration-generated beach morphodynamics in relation to sea turtle nesting behaviour. Int. J. Remote Sens..

[21] Ware M, Long JW, Fuentes MM (2019). Using wave runup modeling to inform coastal species management:an example application for sea turtle nest relocation. Ocean Coast. Manag..

[22] Varela MR, Patrício AR, Anderson K, Broderick AC, DeBell L, Hawkes LA, Godley BJ (2019). Assessing climate change associated sea-level rise impacts on sea turtle nesting beaches using drones, photogrammetry and a novel GPS system. Glob. Chang. Biol..

[23] Gracia V, Sierra JP, Gómez M, Pedrol M, Sampé S, García-León M, Gironella X (2019). Assessing the impact of sea level rise on port operability using LiDAR-derived digital elevation models. Remote Sens. Environ..

[24] IUCN. Guidelines for using the IUCN Red List categories and criteria, version 15 (2022).

[25] Limpus CJ, Miller JD, Parmenter CJ, Limpus DJ (2003). The green turtle, *Chelonia mydas*, population of Raine Island and the northern Great Barrier Reef: 1843–2001. Mem. Queensl. Mus..

[26] Church JA, White NJ (2006). A 20th century acceleration in global sea-level rise. Geophys. Res. Lett..

[27] Ibrahim HD, Sun Y (2020). Mechanism study of the 2010–2016 rapid rise of the Caribbean Sea Level. Glob. Planet. Change.

[28] Intergovernmental Panel on Climate Change (IPCC). In *Climate change 2001: The scientific basis. Contribution of Working Group 1 to the Third Assessment Report of the Intergovernmental Panel on Climate Change* (eds Houghton, J. T.* et al*.) 881 (Cambridge University Press, 2001).

[29] Rivas ML, Fernández C, Marco A (2016). Nesting ecology and population trend of leatherback turtles *Dermochelys coriacea* at Pacuare Nature Reserve, Costa Rica. Oryx.

[30] Rivas ML, Tomillo PS, Diéguez-Uribeondo J, Marco A (2016). Potential effects of dune scarps caused by beach erosion on the nesting behavior of leatherback turtles. Mar. Ecol. Prog. Ser..

[31] Esteban N, Laloë JO, Kiggen FS, Ubels SM, Becking LE, Meesters EH, Christianen MJ (2018). Optimism for mitigation of climate warming impacts for sea turtles through nest shading and relocation. Sci. Rep..

[32] Camargo AJ, Álvarez Gutiérrez Y, Jaramillo Véliz J, Sánchez Tortosa F (2020). Nesting failure of sea turtles in Ecuador-causes of the loss of sea turtle nests: The role of the tide. J. Coast. Conserv..

[33] Vousdoukas MI, Ranasinghe R, Mentaschi L, Plomaritis TA, Athanasiou P, Luijendijk A, Feyen L (2020). Sandy coastlines under threat of erosion. Nat. Clim. Change.

[34] Horrocks JA, Stapleton S, Guada H, Lloyd C, Harris E, Fastigi M, Eckert KL (2016). International movements of adult female leatherback turtles in the Caribbean: Results from tag recovery data (2002–2013). Endanger. Species Res..

[35] Snape RT, Broderick AC, Çiçek BA, Fuller WJ, Glen F, Stokes K, Godley BJ (2016). Shelf life: Neritic habitat use of a turtle population highly threatened by fisheries. Divers. Distrib..

[36] Fuentes MM, Pike DA, Dimatteo A, Wallace BP (2013). Resilience of marine turtle regional management units to climate change. Glob. Change Biol..

[37] Hays GC, Mackay A, Adams CR, Mortimer JA, Speakman JR, Boerema M (1995). Nest site selection by sea turtles. J. Mar. Biol. Assoc. UK K..

[38] López-Castro MC, Carmona R, Nichols WJ (2004). Nesting characteristics of the olive ridley turtle (*Lepidochelys olivacea*) in Cabo Pulmo, southern Baja California. Mar. Biol..

[39] Wood DW, Bjorndal KA (2000). Relation of temperature, moisture, salinity, and slope to nest site selection in loggerhead sea turtles. Copeia.

[40] Slater DA, Felikson D, Straneo F, Goelzer H, Little CM, Morlighem M, Nowicki S (2020). Twenty-first century ocean forcing of the Greenland ice sheet for modelling of sea level contribution. Cryosphere.

[41] Pattyn F, Morlighem M (2020). The uncertain future of the Antarctic Ice Sheet. Science.

[42] Clark PU, He F, Golledge NR, Mitrovica JX, Dutton A, Hoffman JS, Dendy S (2020). Oceanic forcing of penultimate deglacial and last interglacial sea-level rise. Nature.

[43] Yamamoto KH, Powell RL, Anderson S, Sutton PC (2012). Using LiDAR to quantify topographic and bathymetric details for sea turtle nesting beaches in Florida. Remote Sens. Environ..

[44] Yamamoto KH, Anderson SJ, Sutton PC (2015). Measuring the effects of morphological changes to sea turtle nesting beaches over time with LiDAR data. J. Sea Res..

[45] Fuentes MM, Allstadt AJ, Ceriani SA, Godfrey MH, Gredzens C, Helmers D, Bateman BL (2020). Potential adaptability of marine turtles to climate change may be hindered by coastal development in the USA. Region. Environ. Change.

[46] Dellert LJ, O'Neil D, Cassill DL (2014). Effects of beach renourishment and clutch relocation on the success of the loggerhead sea turtle (*Caretta caretta*) eggs and hatchlings. J. Herpetol..

[47] Fuentes MM, Fish MR, Maynard JA (2012). Management strategies to mitigate the impacts of climate change on the sea turtle’s terrestrial reproductive phase. Mitig. Adapt. Strateg. Glob. Chang..

[48] Peters GP, Andrew RM, Canadell JG, Friedlingstein P, Jackson RB, Korsbakken JI, Peregon A (2020). Carbon dioxide emissions continue to grow amidst slowly emerging climate policies. Nature Clim. Change.

