# Author Correction: Uncertain future for global sea turtle populations in face of sea level rise

**DOI:** 10.1038/s41598-023-36350-7

**Published:** 2023-06-07

**Authors:** Marga L. Rivas, Emilio Rodríguez‑Caballero, Nicole Esteban, Antonio J. Carpio, Barbara Barrera‑Vilarmau, Mariana M. P. B. Fuentes, Katharine Robertson, Julia Azanza, Yolanda León, Zaida Ortega

**Affiliations:** 1 Biology Department, Marine Research Institute INMAR, University of Cádiz, Cádiz, Spain; 2 Agronomy Department of the University of Almería and Research Centre for Scientific Collections from the University of Almería (CECOUAL), Almería, Spain; 3 Bioscience Department, Swansea University, Wales, SA2 8PP UK; 4 SaBio Research Group, Instituto de Investigación en Recursos Cinegéticos (IREC-CSIC-UCLM-JCCM), Ciudad Real, Spain; 5 Mondonguillo-Laguna Urpiano NGO, Matina, Costa Rica; 6 Marine Turtle Research, Ecology and Conservation Group, Department of Earth Ocean and Atmospheric Science, Florida State University, Tallahassee, FL USA; 7 Department of Environment and Science, Queensland Government, Brisbane, Australia; 8 University of Habana, Habana, Cuba; 9 Technological Institute of Santo Domingo INTEC, Santo Domingo, Dominican Republic; 10 Department of Ecology and Conservation, Federal University of Mato Grosso Do Sul, Campo Grande, Brazil; 11 Department of Zoology, University of Granada, Granada, Spain

Correction to: *Scientific Reports* 10.1038/s41598-023-31467-1, published online 20 April 2023

The Acknowledgements section in the original Article was incomplete.

“We are grateful to all assistants, coordinators and staff for collection of sea turtle nesting data at each study site. We thank the NGOs and Government Departments coordinating long-term conservation monitoring programmes such as Refugio de Vida silvestre Laguna Urpiano, Costa Rica; Grupo Jaragua, Dominican Republic; St Eustatius National Parks; Foundation (STENAPA) and also people as Jessica Berkel and the researcher Julianna Kadar. Zaida Ortega was funded by a PNPD/CAPES postdoctoral fellowship (#1694744). Marga L Rivas and Zaida Ortega were supported by Postdoctoral Research Contracts of the Andalusian government (Spain) and FEDER EU funds (refs. 839 and 401). Antonio J. Carpio is supported by a “Juan de la Cierva” contract (IJC2020-042629-I) funded by MCIN/AEI/10.13039/501100011033 and by the European Union Next Generation EU/PRTR. Emilio Rodriguez-Caballero was supported by Ramon y Cajal fellowship founded by Agencia Estatal de Investigación (RYC2020-030762-I/AEI/10.13039/501100011033). O.A. was supported by the Plan Propio and the Institute of Marine Sciences of the University of Cádiz.”

now reads:

We are grateful to all assistants, coordinators and staff for collection of sea turtle nesting data at each study site. We thank the NGOs and Government Departments coordinating long-term conservation monitoring programmes such as Refugio de Vida silvestre Laguna Urpiano, Costa Rica; Grupo Jaragua, Dominican Republic; St Eustatius National Parks; Foundation (STENAPA) and also people as Jessica Berkel, the researcher Julianna Kadar, and Rebecca N Handcock. Zaida Ortega was funded by a PNPD/CAPES postdoctoral fellowship (#1694744). Marga L Rivas and Zaida Ortega were supported by Postdoctoral Research Contracts of the Andalusian government (Spain) and FEDER EU funds (refs. 839 and 401). Antonio J. Carpio is supported by a “Juan de la Cierva” contract (IJC2020-042629-I) funded by MCIN/AEI/10.13039/501100011033 and by the European Union Next Generation EU/PRTR. Emilio Rodriguez-Caballero was supported by Ramon y Cajal fellowship founded by Agencia Estatal de Investigación (RYC2020-030762-I/AEI/10.13039/501100011033). O.A. was supported by the Plan Propio and the Institute of Marine Sciences of the University of Cádiz.

The original Article has been updated.

