# Supplementary material for: Uncertain future for global sea turtle populations in face of sea level rise
Source: Sci Rep. 2023 Apr 20;13:5277. doi: 10.1038/s41598-023-31467-1 (PMC10119306; doi:10.1038/s41598-023-31467-1)
Supplement: Supplementary file 1 — Supplementary Information. [file 41598_2023_31467_MOESM1_ESM.zip › SI_Guide.docx]

**aSUPPLEMENTARY INFORMATION GUIDE**

**TITLE:** Uncertain future for global sea turtle populations in face of sea level rise

The Supplementary Information contains three files:

- **Supplementary Tables:** this file contains 18 tables providing the full numerical results of the analyses.

**Supplementary Table 1** Elevation, SLR projection and % of nest estimated to flood at each of the seven study areas by each of the 5 DEMs.

**Supplementary Table 2** Logistic regression model on the probability of being flooded at the seven study areas with the five DEMs.

**Supplementary Table 3** Summary of the seven logistic regression models (for each study area) on the probability of being flooded by the five DEMs.

**Supplementary Table 4** Proportion of nest estimated to be flooded at the seven study areas using the CoastalDEM projections.

**Supplementary Table 5** Logistic regression model on the probability of being flooded in Florida using the CoastalDEM projections.

**Supplementary Table 6** Logistic regression model on the probability of being flooded in Cuba using the CoastalDEM projections.

**Supplementary Table 7** Logistic regression model on the probability of being flooded in St Eustatius using the CoastalDEM projections.

**Supplementary Table 8** Candidate models for fitting the probability of being flooded in Costa Rica using the empirical data.

**Supplementary Table 9** Summary of the best model for fitting the probability of being flooded in Costa Rica using the empirical data.

**Supplementary Table 10** Errors of the five DEMs in comparison to the empirical data for Costa Rica.

**Supplementary Table 11** Goodness-of-fit of the logistic regression models fitting the probability of being flooded in Costa Rica using the empirical data and the five DEMs.

**Supplementary Table 12** Validation of the logistic regression models fitting the probability of being flooded in Costa Rica using the five DEMs in comparison with the empirical data.

**Supplementary Table 13** Candidate models for fitting the probability of being flooded in Ecuador using the empirical data.

**Supplementary Table 14** Averaged coefficients of the best models for fitting the probability of being flooded in Ecuador using the empirical data.

**Supplementary Table 15** Summary of the model fitting the probability of being flooded in Ecuador (using the empirical data) by species.

**Supplementary Table 16** Errors of the five DEMs in comparison to the empirical data for Ecuador.

**Supplementary Table 17** Goodness-of-fit of the logistic regression models fitting the probability of being flooded in Ecuador using the empirical data and the five DEMs.

**Supplementary Table 18** Validation of the logistic regression models fitting the probability of being flooded in Ecuador using the five DEMs in comparison with the empirical data.

- **Supplementary Results:** this file contains the results of the validation of the DEMs of Costa Rica and Ecuador with the empirical data of slope and elevation.
- **Supplementary File:** Locations of marine turtle’s nests georeferenced in the CoastalDEM maps for 2050 and 2100. It is a KMZ file that can be easily visualized on Google Earth software.
