# Supplementary material for: Uncertain future for global sea turtle populations in face of sea level rise
Source: Sci Rep. 2023 Apr 20;13:5277. doi: 10.1038/s41598-023-31467-1 (PMC10119306; doi:10.1038/s41598-023-31467-1)
Supplement: Supplementary file 1 — Supplementary Information. [file 41598_2023_31467_MOESM1_ESM.zip › Supplementary_Tables.docx]

**SUPPLEMENTARY TABLES**

**TITLE:** Uncertain future for global sea turtle populations in face of sea level rise

**Supplementary Table 1.** Descriptive statistics of the current elevation estimated by each DEM at seven turtle rookeries (in m), the mean SLR projected by the IPCC (in m) and the mean % of nests that would be flooded (i.e., SLR > estimated elevation of the nest) according to mean SLR.

| **Area** | **Nests (n)** | **Mean SLR** | **SE SLR** | **DEM** | **Mean elevation** | **SE elevation** | **Mean % of flooded nests** |
| --- | --- | --- | --- | --- | --- | --- | --- |
| **Florida** | 1750 | 0.41 | <0.01 | **also** | 2.07 | 0.04 | 16.00 |
|  |  |  |  | **aster** | 1.69 | 0.04 | 29.60 |
|  |  |  |  | **hydrosheds** | 0.04 | 0.01 | 88.00 |
|  |  |  |  | **srtm30** | 2.59 | 0.05 | 16.00 |
|  |  |  |  | **srtm90** | 1.62 | 0.05 | 42.40 |
| **Cuba** | 2282 | 0.39 | <0.01 | **alos** | 5.29 | 0.04 | 1.23 |
|  |  |  | 0.00 | **aster** | 3.23 | 0.06 | 19.02 |
|  |  |  | 0.00 | **hydrosheds** | 2.51 | 0.07 | 28.22 |
|  |  |  | 0.00 | **srtm30** | 9.74 | 0.05 | 1.23 |
|  |  |  | 0.00 | **srtm90** | 7.25 | 0.08 | 9.82 |
| **Dominican Republic** | 12754 | 0.36 | <0.01 | **alos** | 0.07 | 0.00 | 93.19 |
|  |  |  | 0.00 | **aster** | 2.39 | 0.03 | 38.09 |
|  |  |  | 0.00 | **hydrosheds** | -1.14 | 0.02 | 96.27 |
|  |  |  | 0.00 | **srtm30** | 0.65 | 0.02 | 56.64 |
|  |  |  | 0.00 | **srtm90** | 0.26 | 0.01 | 84.19 |
| **Costa Rica** | 15498 | 0.36 | <0.01 | **alos** | 0.29 | 0.01 | 79.49 |
|  |  |  | 0.00 | **aster** | 15.17 | 0.03 | 0.00 |
|  |  |  | 0.00 | **hydrosheds** | -3.00 | 0.06 | 58.99 |
|  |  |  | 0.00 | **srtm30** | 3.00 | 0.02 | 42.55 |
|  |  |  | 0.00 | **srtm90** | 7.95 | 0.03 | 2.71 |
| **Ecuador** | 2674 | 0.31 | <0.01 | **alos** | 1.34 | 0.11 | 86.91 |
|  |  |  | 0.00 | **aster** | 18.35 | 0.18 | 0.00 |
|  |  |  | 0.00 | **hydrosheds** | 1.49 | 0.10 | 88.48 |
|  |  |  | 0.00 | **srtm30** | 4.41 | 0.22 | 83.25 |
|  |  |  | 0.00 | **srtm90** | 2.66 | 0.15 | 86.91 |
| **St Eustatius** | 3052 | 0.36 | <0.01 | **alos** | 7.46 | 0.08 | 11.01 |
|  |  |  | 0.00 | **aster** | 11.72 | 0.07 | 0.92 |
|  |  |  | 0.00 | **hydrosheds** | 6.94 | 0.08 | 6.42 |
|  |  |  | 0.00 | **srtm30** | 6.56 | 0.08 | 12.39 |
|  |  |  | 0.00 | **srtm90** | 12.89 | 0.12 | 3.21 |
| **Australia** | 1470 | 0.37 | 0.01 | **alos** | 5.96 | 0.03 | 0.00 |
|  |  |  | 0.01 | **aster** | 0.00 | 0.00 | 100.00 |
|  |  |  | 0.01 | **hydrosheds** | 1.71 | 0.03 | 26.67 |
|  |  |  | 0.01 | **srtm30** | 5.68 | 0.03 | 0.95 |
|  |  |  | 0.01 | **srtm90** | 4.70 | 0.07 | 20.95 |

**Supplementary Table 2.** Summary of the logistic regression model on the probability of flooding by study area, the DEM, and the interaction between RCP and year of projection as fixed terms**.**

| **Term** | **Coefficient** | **SE** | **F** | **P** |
| --- | --- | --- | --- | --- |
| (Intercept) | 0.670 | 0.038 | 17.708 | **<0.001** |
| Area: Cuba | -1.809 | 0.042 | -42.643 | **<0.001** |
| Area: Dominican Republic | 1.952 | 0.030 | 66.154 | **<0.001** |
| Area: Costa Rica | -0.094 | 0.028 | -3.300 | **0.001** |
| Area: Ecuador | 1.650 | 0.036 | 46.412 | **<0.001** |
| Area: St Eustatius | -2.465 | 0.045 | -55.182 | **<0.001** |
| Area: Australia | -0.504 | 0.041 | -12.326 | **<0.001** |
| DEM: aster | -3.064 | 0.022 | -138.641 | **<0.001** |
| DEM: hydrosheds | -0.102 | 0.019 | -5.252 | **<0.001** |
| DEM: srtm30 | -1.520 | 0.019 | -78.542 | **<0.001** |
| DEM: srtm90 | -1.797 | 0.020 | -91.476 | **<0.001** |
| RCP: RCP8.5 | -0.012 | 0.035 | -0.342 | 0.732 |
| Year projection: 2040 | 0.009 | 0.035 | 0.264 | 0.792 |
| Year projection: 2050 | 0.003 | 0.035 | 0.072 | 0.943 |
| Year projection: 2060 | -0.002 | 0.035 | -0.048 | 0.962 |
| Year projection: 2070 | -0.013 | 0.035 | -0.364 | 0.716 |
| Year projection: 2080 | -0.028 | 0.035 | -0.809 | 0.418 |
| Year projection: 2090 | 0.000 | 0.035 | 0.000 | 1.000 |
| Year projection: 2100 | -0.006 | 0.035 | -0.161 | 0.872 |
| RCP8.5 ｘ year projection 2040 | 0.005 | 0.049 | 0.105 | 0.917 |
| RCP8.5 ｘ year projection 2050 | 0.009 | 0.049 | 0.192 | 0.847 |
| RCP8.5 ｘ year projection 2060 | 0.005 | 0.049 | 0.100 | 0.921 |
| RCP8.5 ｘ year projection 2070 | 0.007 | 0.049 | 0.152 | 0.879 |
| RCP8.5 ｘ year projection 2080 | 0.059 | 0.049 | 1.195 | 0.232 |
| RCP8.5 ｘ year projection 2090 | 0.017 | 0.049 | 0.344 | 0.731 |
| RCP8.5 ｘ year projection 2100 | -0.017 | 0.049 | -0.337 | 0.736 |

**Supplementary Table 3.** Summary of the individual logistic regression models fitted for each of the seven study areas. For each area, the model with the lowest AICc was selected.

| **Area** | **Term** | **Coefficient** | **SE** | **F** | **P** |
| --- | --- | --- | --- | --- | --- |
| **Florida** | **Flooded ~ DEM + RCP + year projection** | | | | |
|  | Intercept | -1.658 | 0.093 | -17.926 | **<0.001** |
|  | DEM: aster | 0.792 | 0.078 | 10.122 | **<0.001** |
|  | DEM: hydrosheds | 3.651 | 0.092 | 39.702 | **<0.001** |
|  | DEM: srtm30 | 0.000 | 0.086 | 0.000 | 1.000 |
|  | DEM: srtm90 | 1.352 | 0.076 | 17.801 | **<0.001** |
|  | RCP: RCP8.5 | 0.000 | 0.049 | 0.000 | 1.000 |
|  | Year projection: 2040 | 0.000 | 0.098 | 0.000 | 1.000 |
|  | Year projection: 2050 | 0.000 | 0.098 | 0.000 | 1.000 |
|  | Year projection: 2060 | 0.000 | 0.098 | 0.000 | 1.000 |
|  | Year projection: 2070 | 0.000 | 0.098 | 0.000 | 1.000 |
|  | Year projection: 2080 | 0.000 | 0.098 | 0.000 | 1.000 |
|  | Year projection: 2090 | 0.000 | 0.098 | 0.000 | 1.000 |
|  | Year projection: 2100 | 0.000 | 0.098 | 0.000 | 1.000 |
| **Cuba** | **Flooded ~ DEM + RCP + year projection** | | | | |
|  | Intercept | -4.388 | 0.195 | -22.460 | **<0.001** |
|  | DEM: aster | 2.939 | 0.185 | 15.912 | **<0.001** |
|  | DEM: hydrosheds | 3.455 | 0.183 | 18.866 | **<0.001** |
|  | DEM: srtm30 | 0.000 | 0.252 | 0.000 | 1.000 |
|  | DEM: srtm90 | 2.170 | 0.190 | 11.444 | **<0.001** |
|  | RCP: RCP8.5 | 0.000 | 0.057 | 0.000 | 1.000 |
|  | Year projection: 2040 | 0.000 | 0.114 | 0.000 | 1.000 |
|  | Year projection: 2050 | 0.000 | 0.114 | 0.000 | 1.000 |
|  | Year projection: 2060 | 0.000 | 0.114 | 0.000 | 1.000 |
|  | Year projection: 2070 | 0.000 | 0.114 | 0.000 | 1.000 |
|  | Year projection: 2080 | 0.000 | 0.114 | 0.000 | 1.000 |
|  | Year projection: 2090 | 0.000 | 0.114 | 0.000 | 1.000 |
|  | Year projection: 2100 | 0.000 | 0.114 | 0.000 | 1.000 |
| **Dominican Republic** | **Flooded ~ DEM + species** | | | | |
|  | Intercept | 2.134 | 0.044 | 48.883 | **<0.001** |
|  | DEM: aster | -3.118 | 0.037 | -83.922 | **<0.001** |
|  | DEM: hydrosheds | 0.634 | 0.055 | 11.592 | **<0.001** |
|  | DEM: srtm30 | -2.361 | 0.037 | -63.841 | **<0.001** |
|  | DEM: srtm90 | -0.947 | 0.040 | -23.668 | **<0.001** |
|  | Species: hawksbill | 0.540 | 0.033 | 16.276 | **<0.001** |
|  | Species: leatherback | 0.637 | 0.065 | 9.797 | **<0.001** |
| **Costa Rica** | **Flooded ~ DEM + RCP + year projection** | | | | |
|  | Intercept | 1.355 | 0.032 | 42.535 | **<0.001** |
|  | DEM: aster | -19.921 | 49.011 | -0.406 | 0.684 |
|  | DEM: hydrosheds | -0.991 | 0.024 | -41.179 | **<0.001** |
|  | DEM: srtm30 | -1.655 | 0.024 | -68.892 | **<0.001** |
|  | DEM: srtm90 | -4.936 | 0.050 | -98.958 | **<0.001** |
|  | RCP: RCP8.5 | 0.000 | 0.018 | 0.000 | 1.000 |
|  | Year projection: 2040 | 0.000 | 0.037 | 0.000 | 1.000 |
|  | Year projection: 2050 | 0.000 | 0.037 | 0.000 | 1.000 |
|  | Year projection: 2060 | 0.000 | 0.037 | 0.000 | 1.000 |
|  | Year projection: 2070 | 0.000 | 0.037 | 0.000 | 1.000 |
|  | Year projection: 2080 | 0.000 | 0.037 | 0.000 | 1.000 |
|  | Year projection: 2090 | 0.000 | 0.037 | 0.000 | 1.000 |
|  | Year projection: 2100 | 0.000 | 0.037 | 0.000 | 1.000 |
| **Ecuador** | **Flooded ~ DEM + species** | | | | |
|  | Intercept | 1.822 | 0.062 | 29.377 | **<0.001** |
|  | DEM: aster | -20.463 | 117.939 | -0.174 | 0.862 |
|  | DEM: hydrosheds | 0.146 | 0.078 | 1.869 | 0.062 |
|  | DEM: srtm30 | -0.290 | 0.072 | -4.013 | **<0.001** |
|  | DEM: srtm90 | 0.000 | 0.076 | 0.000 | 1.000 |
|  | Species: hawksbill | 0.138 | 0.055 | 2.513 | 0.012 |
|  | Species: olive | -0.042 | 0.118 | -0.356 | 0.722 |
| **St Eustatius** | **Flooded ~ DEM + species** | | | | |
|  | Intercept | -2.485 | 0.065 | -38.527 | **<0.001** |
|  | DEM: aster | -2.571 | 0.186 | -13.794 | **<0.001** |
|  | DEM: hydrosheds | -0.553 | 0.089 | -6.194 | **<0.001** |
|  | DEM: srtm30 | 0.186 | 0.076 | 2.435 | 0.015 |
|  | DEM: srtm90 | -1.289 | 0.111 | -11.560 | **<0.001** |
|  | Species: hawksbill | 1.100 | 0.071 | 15.425 | **<0.001** |
|  | Species: leatherback | 0.505 | 0.082 | 6.164 | **<0.001** |
| **Australia** | **Flooded ~ DEM + RCP + year projection** | | | | |
|  | Intercept | -19.566 | 262.371 | -0.075 | 0.941 |
|  | DEM: aster | 39.132 | 371.049 | 0.105 | 0.916 |
|  | DEM: hydrosheds | 18.554 | 262.371 | 0.071 | 0.944 |
|  | DEM: srtm30 | 14.922 | 262.371 | 0.057 | 0.955 |
|  | DEM: srtm90 | 18.238 | 262.371 | 0.070 | 0.945 |
|  | RCP: RCP8.5 | 0.000 | 0.080 | 0.000 | 1.000 |
|  | Year projection: 2040 | 0.000 | 0.160 | 0.000 | 1.000 |
|  | Year projection: 2050 | 0.000 | 0.160 | 0.000 | 1.000 |
|  | Year projection: 2060 | 0.000 | 0.160 | 0.000 | 1.000 |
|  | Year projection: 2070 | 0.000 | 0.160 | 0.000 | 1.000 |
|  | Year projection: 2080 | 0.000 | 0.160 | 0.000 | 1.000 |
|  | Year projection: 2090 | 0.000 | 0.160 | 0.000 | 1.000 |
|  | Year projection: 2100 | 0.000 | 0.160 | 0.000 | 1.000 |

**Supplementary Table 4**. Proportion of flooded nests per rookery in moderate scenarios of sea level rise (Kopp et al. 2014) for 2050 and 2100 according to the Climate Central CoastalDEM maps at each study area.

| **Area** | **Nests (n)** | **Flooded nests 2050 (%)** | **Flooded nests 2100 (%)** |
| --- | --- | --- | --- |
| Raine Island (Australia) | 105 | 100.00 | 100.00 |
| Mondonguillo beach (Costa Rica) | 1107 | 99.91 | 100.00 |
| Guanahacabibes Peninsula (Cuba) | 163 | 8.59 | 8.59 |
| Saona Island (Dominican Republic) | 911 | 100.00 | 100.00 |
| Seven Pacific beaches (Ecuador) | 207 | 89.86 | 91.30 |
| St George Island (USA) | 124 | 28.23 | 73.39 |
| Atlantic & Caribbean beaches (St Eustatius) | 218 | 20.18 | 20.64 |

**Supplementary Table 5**. Results of the logistic regression model on the probability of being flooded in Florida, estimated by the CoastalDEM data for the RCP 4.5 and the moderate SLR scenario (Kopp. et al. 2014), with the year of projection (2050 vs 2100) as fixed factor. AIC = 222.490

| Term | Coefficient | SE | F | P |
| --- | --- | --- | --- | --- |
| (Intercept) | -0.996 | 0.239 | -4.171 | **< 0.001** |
| Year (2100) | 1.949 | 0.329 | 5.920 | **< 0.001** |

**Supplementary Table 6**. Results of the logistic regression model on the probability of being flooded in Cuba, estimated by the CoastalDEM data for the RCP 4.5 and the moderate SLR scenario (Kopp. et al. 2014), with the year of projection (2050 vs 2100) as fixed factor. AIC = 147.268

| Term | Coefficient | SE | F | P |
| --- | --- | --- | --- | --- |
| (Intercept) | -2.425 | 0.330 | -7.350 | **< 0.001** |
| Year (2100) | 0.113 | 0.457 | 0.248 | 0.804 |

**Supplementary Table 7**. Results of the logistic regression model on the probability of being flooded in St Eustatius, estimated by the CoastalDEM data for the RCP 4.5 and the moderate SLR scenario (Kopp. et al. 2014), with the year of projection (2050 vs 2100) and the species (green, hawksbill and leatherback) as fixed factors. AIC = 303.355

| Term | estimate | std.error | statistic | p.value |
| --- | --- | --- | --- | --- |
| (Intercept) | -1.883 | 0.250 | -7.528 | **< 0.001** |
| Year (2100) | -0.017 | 0.293 | -0.058 | 0.954 |
| Species (hawksbill) | 0.388 | 0.406 | 0.955 | 0.339 |
| Species (leatherback) | 1.892 | 0.331 | 5.722 | 0.000 |

**Supplementary Table 8** Set of candidate models for fitting the probability of being flooded in Costa Rica (N = 95 nests, leatherback turtle) using the empirical data (slope of the beach and distance to the water manually registered for each nest location) and the IPCC SLR projections (2030-2100, by decade; RCP 4.5 and RCP 8.5). The initial logistic regression model includes the RCP, the year of projection and its interaction term.

| **(Intercept)** | **Year** | **RCP** | **Year ｘ RCP** | **df** | **logLik** | **AICc** | **ΔAICc** | **w** |
| --- | --- | --- | --- | --- | --- | --- | --- | --- |
| -1.032 | + | 0.201 |  | 9 | -737 | 1493 | 0.000 | 0.568 |
| -1.028 | + |  |  | 8 | -739 | 1494 | 0.595 | 0.422 |
| -1.030 | + | 0.0991 | + | 16 | -734 | 1501 | 7.965 | 0.011 |
| -0.403 |  | 0.1827 |  | 2 | -766 | 1537 | 43.972 | 0 |
| -0.402 |  |  |  | 1 | -768 | 1537 | 44.247 | 0 |

**Supplementary Table 9.** Summary of the best model selected for fitting the probability of being flooded in Costa Rica (N = 95 nests, leatherback turtle) using the empirical data (slope of the beach and distance to the water manually registered for each nest location) and the IPCC SLR projections (2030-2100, by decade; RCP 4.5 and RCP 8.5). From the candidate models of Table S8, only this model (including the additive effects of the year and the RCP) has ΔAICc < 2 and an accumulated Akaike’s weight up to 0.95.

|  | **Coefficient** | **SE** | **Exponentiated coeff.** | **Z** | **p** |
| --- | --- | --- | --- | --- | --- |
| Intercept | -1.135 | 0.199 | 0.321 | -5.713 | **<0.001** |
| Year: 2040 | 0.062 | 0.265 | 0.940 | -0.232 | 0.817 |
| Year: 2050 | 0.444 | 0.256 | 1.558 | 1.734 | 0.083 |
| Year: 2060 | 0.460 | 0.255 | 1.586 | 1.807 | 0.071 |
| Year: 2070 | 0.712 | 0.253 | 2.039 | 2.810 | **0.005** |
| Year: 2080 | 0.971 | 0.259 | 2.641 | 3.744 | **<0.001** |
| Year: 2090 | 1.143 | 0.251 | 3.136 | 4.560 | **<0.001** |
| Year: 2100 | 1.283 | 0.251 | 3.606 | 5.118 | **<0.001** |
| RCP: 8.5 | 0.201 | 0.124 | 1.223 | 1.619 | 0.105 |

χ²(10) = 86.365, p = 0.000

Pseudo-R² (Cragg-Uhler) = 0.070

Pseudo-R² (McFadden) = 0.039

AIC = 1492.871, BIC = 1538.220

Standard errors: MLE

**Supplementary Table 10** Summary of the errors (real value from empirical data - value estimated by the correspondent DEM) of the five DEMs regarding the slope and elevation of each nest location in Costa Rica.

|  | **Slope (radians)** | | | | **Elevation (m)** | | | |
| --- | --- | --- | --- | --- | --- | --- | --- | --- |
|  | **Estimations** | | **Error** | | **Estimations** | | **Error** | |
|  | **mean** | **sd** | **mean** | **sd** | **mean** | **sd** | **mean** | **sd** |
| Empirical data | 0.040 | 0.042 | - | - | 0.710 | 0.744 | **-** | **-** |
| alos | 0.008 | 0.014 | 0.032 | 0.047 | 0.168 | 0.387 | 0.541 | 0.905 |
| aster | 0.071 | 0.019 | -0.031 | 0.047 | 1.325 | 0.840 | -0.630 | 0.845 |
| hydrosheds | 0.029 | 0.020 | 0.011 | 0.037 | 0.531 | 0.561 | 0.174 | 0.628 |
| srtm30 | 0.0451 | 0.037 | -0.005 | 0.060 | 0.832 | 0.815 | -0.131 | 1.183 |
| srtm90 | 0.042 | 0.019 | -0.002 | 0.050 | 0.788 | 0.618 | -0.087 | 0.793 |

**Supplementary Table 11.** Goodness-of-fit quality of the different models using the empirical data or any of the six DEMs. All models include the RCP and the year of projection (2030-2100, by decade) as fixed factors (flooded ~ Year + RCP). Degrees of freedom are 1127 for all models.

| **Model** | **Null deviance** | **Pseudo-R2 (Cragg-Uhler)** | **Pseudo-R2 (McFadden)** | **AICc** | **BIC** |
| --- | --- | --- | --- | --- | --- |
| Empirical data | 1522.9 | 0.068 | 0.038 | 1482.9 | 1528.2 |
| ALOS 30m | 884.4 | 0.079 | 0.056 | 852.6 | 897.9 |
| ASTER 100m | 530.1 | 0.140 | 0.114 | 487.5 | 532.7 |
| HydroSHEDS 90m | 1542.6 | 0.239 | 0.143 | 1339.3 | 1384.5 |
| SRTM 30m | 1495.5 | 0.067 | 0.038 | 1456.9 | 1502.2 |
| SRTM 90m | 1259.8 | 0.205 | 0.133 | 1110.8 | 1156.1 |

**Supplementary Table 12**. Validation of the models of the five DEMs with the empirical data, that is, whether nests would be flooded using the *in situ* measurements of slope and distance to the sea. The column Kendall provides the Kendall correlation coefficient for paired data, pairing each prediction of each nest at each DEM with the prediction of the same nest in the empirical data. The column Kappa includes results of the Cohen’s kappa coefficient.

| **DEM** | **Sensitivity** | **Specificity** | **Accuracy** | **Kendall** | **Kappa** |
| --- | --- | --- | --- | --- | --- |
| alos | 38.46 | 45.99 | 39.46 | -0.108 | -0.063 |
| aster | 20.80 | 57.73 | 55.31 | -0.108 | -0.062 |
| hydrosheds | 46.25 | 63.20 | 55.91 | 0.095 | 0.095 |
| srtm30 | 33.56 | 54.74 | 46.77 | -0.115 | -0.115 |
| srtm90 | 40.23 | 58.93 | 54.30 | -0.007 | -0.007 |

**Supplementary Table 13** Set of candidate models for fitting the probability of being flooded in Ecuador (N = 85 nests, green, hawksbill and olive turtles) using the empirical data (slope of the beach and distance to the water manually registered for each nest location) and the IPCC SLR projections (2030-2100, by decade; RCP 4.5 and RCP 8.5). The initial logistic regression model includes the RCP, the year of projection, the species and all their interaction terms.

| **(Intercept)** | **Year** | **RCP** | **Sp** | **Year**  **ｘRCP** | **Year**  **ｘSp** | **RCP**  **ｘSp** | **YearｘRCPｘSp** | **df** | **logLik** | **AICc** | **delta** | **w** |
| --- | --- | --- | --- | --- | --- | --- | --- | --- | --- | --- | --- | --- |
| -3.760 |  |  | -1.324 |  |  |  |  | 2 | -122.473 | 248.957 | 0.000 | 0.626 |
| -3.762 |  | 0.127 | -1.323 |  |  |  |  | 3 | -122.419 | 250.862 | 1.905 | 0.241 |
| -3.768 |  | -0.025 | -1.314 |  |  | -0.515 |  | 4 | -122.207 | 252.453 | 3.495 | 0.109 |
| -3.872 | + |  | -1.325 |  |  |  |  | 9 | -119.451 | 257.081 | 8.123 | 0.011 |
| -3.568 |  |  |  |  |  |  |  | 1 | -128.282 | 258.568 | 9.611 | 0.005 |
| -3.877 | + | 0.122 | -1.323 |  |  |  |  | 10 | -119.402 | 259.021 | 10.064 | 0.004 |
| -3.570 |  | 0.139 |  |  |  |  |  | 2 | -128.216 | 260.445 | 11.487 | 0.002 |
| -3.887 | + | -0.017 | -1.314 |  |  | -0.480 |  | 11 | -119.219 | 260.700 | 11.742 | 0.002 |
| -3.691 | + |  | -0.194 |  | + |  |  | 16 | -116.997 | 266.537 | 17.580 | < 0.001 |
| -3.689 | + |  |  |  |  |  |  | 8 | -125.228 | 266.598 | 17.640 | < 0.001 |
| -3.693 | + | 0.147 |  |  |  |  |  | 9 | -125.155 | 268.489 | 19.531 | < 0.001 |
| -3.694 | + | 0.120 | -0.187 |  | + |  |  | 17 | -116.950 | 268.510 | 19.553 | < 0.001 |
| -3.704 | + | -0.024 | -0.169 |  | + | -0.501 |  | 18 | -116.751 | 270.186 | 21.229 | < 0.001 |
| -3.932 | + | -0.847 | -1.332 | + |  |  |  | 17 | -118.257 | 271.125 | 22.168 | < 0.001 |
| -3.945 | + | -0.982 | -1.324 | + |  | -0.472 |  | 18 | -118.081 | 272.845 | 23.888 | < 0.001 |
| -3.749 | + | -0.760 |  | + |  |  |  | 16 | -124.080 | 280.702 | 31.745 | < 0.001 |
| -3.753 | + | -0.773 | -0.241 | + | + |  |  | 24 | -115.847 | 280.900 | 31.943 | < 0.001 |
| -3.759 | + | -0.786 | -0.274 | + | + | -0.236 |  | 25 | -115.810 | 282.927 | 33.970 | < 0.001 |
| -6.126 | + | -5.341 | 7.923 | + | + | 17.882 | + | 32 | -112.354 | 290.849 | 41.891 | < 0.001 |

**Supplementary Table 14.** Results for the averaged coefficients of the two best models for the empirical data of Ecuador (N = 85 nests, green, hawksbill and olive turtles) using the empirical data (slope of the beach and distance to the water manually registered for each nest location) and the IPCC SLR projections (2030-2100, by decade; RCP 4.5 and RCP 8.5). From the candidate models of Table S13, two models (the first one including only the species, and the second one including the additive effects of the RCP and the species) have ΔAICc < 2 and an accumulated Akaike’s weight up to 0.95.

|  | **Coefficient** | **SE** | **Exponentiated coeff.** | **Z** | **p** |
| --- | --- | --- | --- | --- | --- |
| Intercept | -3.761 | 0.226 | 0.023 | 16.651 | **< 0.001** |
| Species | -1.324 | 0.393 | 0.266 | 3.363 | **< 0.001** |
| RCP | 0.127 | 0.387 | 1.135 | 0.327 | 0.744 |

**Supplementary Table 15.** Results for the probability of being flooded for Ecuador (N = 85 nests, green, hawksbill and olive turtles) using the empirical data (slope of the beach and distance to the water manually registered for each nest location) and the IPCC SLR projections only including the species as fixed factor (the best model of Table 13).

|  | **Coefficient** | **SE** | **Exponentiated coeff.** | **Z** | **p** |
| --- | --- | --- | --- | --- | --- |
| Intercept | -2.833 | 0.250 | 0.059 | -11.535 | < 0.001 |
| Species: hawksbill | -1.324 | 0.393 | 0.266 | -3.363 | < 0.001 |

χ²(1) = 11.618, p = 0.001

Pseudo-R² (Cragg-Uhler) = 0.051

Pseudo-R² (McFadden) = 0.045

AIC = 248.946, BIC = 258.801

**Supplementary Table 16** Summary of the errors (real value from empirical data - value estimated by the correspondent DEM) of the five DEMs regarding the slope and elevation of each nest location in Ecuador.

|  | **Slope (radians)** | | | | **Elevation (m)** | | | |
| --- | --- | --- | --- | --- | --- | --- | --- | --- |
|  | **Estimations** | | **Error** | | **Estimations** | | **Error** | |
|  | **mean** | **sd** | **mean** | **sd** | **mean** | **sd** | **mean** | **sd** |
| Empirical data | 0.168 | 0.099 | - | - | 12.465 | 10.687 | **-** | **-** |
| alos | 0.095 | 0.214 | 0.040 | 0.194 | 3.411 | 8.124 | 9.054 | 9.159 |
| aster | 0.135 | 0.084 | 0.041 | 0.080 | 4.882 | 3.852 | 7.583 | 8.196 |
| hydrosheds | 0.069 | 0.077 | 0.081 | 0.070 | 2.335 | 2.583 | 10.129 | 9.140 |
| srtm30 | 0.126 | 0.214 | 0.006 | 0.174 | 5.094 | 10.093 | 7.371 | 7.205 |
| srtm90 | 0.109 | 0.102 | 0.046 | 0.075 | 3.776 | 3.514 | 8.689 | 8.622 |

**Supplementary** **Table 17.** Goodness-of-fit quality of the different models using the empirical data or any of the six DEMs for Ecuador. All models include the RCP and the year of projection (2030-2100, by decade) as fixed factors (flooded ~ Year + RCP ). Degrees of freedom are 1019 for all models.

| **Model** | **Null deviance** | **Pseudo-R2 (Cragg-Uhler)** | **Pseudo-R2**  **(McFadden)** | **AICc** | **BIC** |
| --- | --- | --- | --- | --- | --- |
| Empirical data | 256.6 | 0.077 | 0.069 | 256.9 | 301.3 |
| ALOS 30m | 1208.5 | 0.122 | 0.075 | 1138.3 | 1187.6 |
| ASTER 100m | 456.4 | 0.068 | 0.056 | 450.9 | 500.2 |
| HydroSHEDS 90m | 1404.6 | 0.012 | 0.007 | 1415.4 | 1464.7 |
| SRTM 30m | 1256.2 | 0.020 | 0.011 | 1261.7 | 1311.0 |
| SRTM 90m | 1113.0 | 0.002 | 0.001 | 1132.0 | 1181.3 |

**Supplementary Table 18**. Validation of the models of the five DEMs with the empirical data of Ecuador. The column Kendall provides the Kendall correlation coefficient for paired data, pairing each prediction of each nest at each DEM with the prediction of the same nest in the empirical data. The column Kappa includes results of the Cohen’s kappa coefficient.

| **DEM** | **Sensitivity** | **Specificity** | **Accuracy** | **Kendall** | **Kappa** |
| --- | --- | --- | --- | --- | --- |
| alos | 0 | 100 | 97.35 | -0.060 | -0.012 |
| aster | 0 | 100 | 97.35 | -0.041 | -0.038 |
| hydrosheds | 22.22 | 84.29 | 82.65 | -0.113 | -0.040 |
| srtm30 | 100 | 0 | 2.65 | 0.030 | 0.006 |
| srtm90 | 100 | 0 | 2.65 | 0.038 | 0.021 |
