# Supplementary material for: Uncertain future for global sea turtle populations in face of sea level rise
Source: Sci Rep. 2023 Apr 20;13:5277. doi: 10.1038/s41598-023-31467-1 (PMC10119306; doi:10.1038/s41598-023-31467-1)
Supplement: Supplementary file 1 — Supplementary Information. [file 41598_2023_31467_MOESM1_ESM.zip › Supplementary info.doc]

**Supplementary Information**

Study areas and species

Sea turtles use temperate and tropical beaches to lay their eggs^51^. The Central America coasts host some of the most important sea turtle nesting rookeries across the world^51,52^ and Australia contains the largest rookery for green turtles^53^. In addition, the characteristics of the beaches studied are very different. For instance, the beaches of St Eustatius and Cuba are narrow and steep, but those of the Dominican, Costa Rican, Floridan and Raine Island are wide and flat. Sea turtles exhibit natal philopatry, returning to the region where they were born^9^, with exceptionally high precision rookeries^10.^ We assessed the vulnerability of five species (leatherback, green, olive ridley, loggerhead and hawksbill) of sea turtle at seven nesting beaches (St George Island, Florida; Guanacabibes, Cuba; Saona Island, Dominican Republic; Costa Rica beach, Costa Rica; St Eustatius beaches; Raine Island, Australia; Ecuador beaches) to SLR (Extended data Table 1 Fig 1).

***Sea-level projections and nest location flooding estimations***

Nest locations were recorded with GPS (typically Garmin Etrex 10 which are accurate to 10 m) in Costa Rica in 2020 and in Ecuador in the 2018-2020 nesting seasons. We estimated whether each nest would be flooded under three different sources of SLR projections: (i) GPS nest locations + DEMs + IPCC projections for the seven studied areas across the globe, (ii) in situ measurements + DEMs + IPCC projections for Costa Rica and Ecuador were collected to validate the accuracy of DEM models with empirical data, and (iii) GPS nest locations were georeferenced in CoastalDEM maps as an alternative low-cost reliable estimation of nest vulnerability to flooding for the seven study areas.

***i)* GPS nest locations + DEMs + IPCC projections**

For the first set of flooding estimations, we extracted nest elevation measurements from five DEMs (the only available ones): 1) “alos 30 m” (Advanced Land Observing Satellite. World 3D – 30 m obtained by radar ALOS PALSAR^54^, 2) “hydrosheds 90 m” (HydroSHEDS ^55^ based on NASA's Shuttle Radar Topography Mission or SRTM), 3) “srtm 30 m” (NASA’s SRTM digital elevation 30 m obtained by The Shuttle Radar Topography Mission V3^56^, 4) “srtm 90 m” (NASA’s SRTM digital elevation 90 m obtained by The Shuttle Radar Topography Mission V4^57^ and 5) “aster 100 m” (Advanced Spaceborn Thermal Emission and Reflection Radiometer) Global Digital Elevation Model V002^58^. Selected DEMs are the highest resolution open-source models available for the rookery location, for example Radar Topography Mission includes vertical resolution at decimeter scale^3^ and were chosen because they are global in nature and would ensure that all 7 study areas were addressed equally. These models have been used at 1 m scale in the United States to construct open coast regions^59^ and at 1-4 m scale in China^60^, however, remote islands and undeveloped coasts remain unrepresented.

Then, we added the SLR projections of the IPCC for the two RCPs for which time series projections are available: the intermediate scenario (RCP 4.5) and the high emissions scenario (RCP 8.5)^41^These data, distributed in netCDF format by the Integrated Climate Data Centre (ICDC) University of Hamburg (Germany), included the total combined mean SLR projections from 2030 to 2100 (by decade) for each nest location (spatial resolution of 1º). We extracted the SLR data and created the full data set with the locations of nests using the R packages ncdf^61^, raster^62^ and rgdal^63^. Whenever the elevation projected by SLR data exceeded the elevation of the nest location estimated by the respective DEM, we assumed that the nest is projected to be flooded (assuming current nest elevations are representative of future nest site selection choices in the respective decade and scenario). This assumption has some limitations as it excludes natural beach morphometrics, beach retreat particularly along undeveloped coastlines and, for example erosion and storm surge during extreme weather events^64^. However, DEMs are currently considered very accurate for estimating the potential effects of SLR on coastal habitats as assessed in other studies^22^.

***ii) in situ measurements + DEMs + IPCC projections***

For the second set of flooding estimations and validation with field data, we computed the altitude of each nest location recorded in Costa Rica (N = 95) and Ecuador in 2020 (N = 85), obtained by trigonometry using the distance from the nest to the mean high water (in meters) and the slope (by clinometer). With these data, we calculated the distance of seawater from the nest sites at each location using each combination of year (by decade) under two emission scenarios. Whenever the distance of seawater inundation onto the beach (estimated as the SLR projection divided by the tangent of the slope) was greater than the 2020 distance between nest and sea, we defined the nest site as flooded. This process defined a binomial variable indicating whether each nest is predicted to be flooded or not using two factors: the year (2010-2100, by decade) and the emissions scenario (RCP 4.5 and RCP 8.5).

To estimate DEM source accuracy, we compared in situ nest height data and the altitude at the nest GPS coordinates obtained from the different DEM sources using the Cohen’s kappa coefficient for each pair of estimations of the empirical vs each DEM with the fmsb R package^65^. We also analysed linear relationships between the mean altitude and slope obtained from different DEM sources and field data.

***iii) GPS nest locations + Coastal DEMs maps + IPCC projections***

For the third set of flooding estimations, we verified the overlap between each current nest location by GPS and georeferenced maps obtained from CoastalDEM (Climate Central; <https://go.climatecentral.org/coastaldem/>), high-accuracy DEM for coastal areas. CoastalDEM as stated by its creators, reduces the median errors of the SRTM DEM to a negligible value, improving the representation of areas at risk of flood (Farr et al., 2007). Top-accuracy DEMs from airborne are unavailable for most of Asia, Africa and Latin America. The free dataset has a 90 m resolution whereas the 30 m resolution maps are available at a higher cost. CoastalDEM maps allow direct visualization of the predicted areas to be flooded from 2030 to 2100 under three emissions scenarios (RCP2.6, RCP4.5, and RCP8.5), and using both the “Moderate”^66^ and the “Pessimistic”^4^ SLR projections. Coastal maps representing flooding areas for 2050 and 2100, according to the SLR moderate scenario^66^ in RCP 4.5, were obtained from the Climate Central. Obtained maps for each of the seven study areas were georeferenced using a set of 50 control points identified by photointerpretation of high spatial resolution images from Esri´s world imagery dataset (<https://www.arcgis.com/home/item.html?id=c1c2090ed8594e0193194b750d0d5f83>). Then,

we combined nest locations and flooded areas maps to classify them into flooded (1) and not flooded nest (0) for five areas (Extended Data Figure 5), excluding Costa Rica and Ecuador due to failure of CoastalDEM® to show the entire coastline of the rookeries at these areas (Extended Data Figure 6).

Models

We fitted analogous machine learning logistic classification models for the three sources of data (DEM + Climate Central maps and empirical measurements + IPCC projections) to calculate the effect of each variable in the probability of areas being flooded and predict the probabilities for each study area (Supplementary Table 4). We used 75% of each dataset to fit the models and the remaining 25% to validate models and make predictions. To calculate accuracy, we classified nests with > 0.5 probability of being flooded as ‘flooded’, indicated as the optimal probability threshold in the Receiver Operating Characteristics (ROC) curves of most models^67,68^. The combination of the main effects of all moderators and their interactions may be potentially modulating the probability of being flooded. Thus, we used a multi-model inference approach using model selection to find the best model (used to calculate the probability predictions) and model averaging (to interpret the effect of moderators in the probability of getting flooded whenever there is model uncertainty). From the set of candidate models, we selected the best models as those with ΔAICc < 2 or with an accumulated Akaike weight of < 95%. For those study areas with multiple nesting species, we also included the species (and interactions with other factors) in the respective models. The models were fitted using the best data available for each study area with available data: empirical data for Ecuador (with the IPCC SLR projections for RCP 4.5 and 8.5 from 2030 to 2100) and CoastalDEM data for St Eustatius and the Dominican Republic (moderate scenario) (Kulp & Strauss, 2018), RCP 4.5 and the projections for 2050 and 2100, as above; see SI). All analyses were conducted in R^69,70^, using the packages CaTools^71^ for ML classification, lme4^72^ for model fitting and MuMIn for model selection and averaging. We assessed the performance of the selected models by fitting them on the 25% of the dataset reserved for validation. Then we compared predictions from the empirical data and the DEMs data. Whenever we had in situ data (Costa Rica and Ecuador), we used the predictions obtained from these empirical data to validate the predictions of each DEM source; for the rest of the models, we used the same type of estimations of those used to fit the model.

To evaluate the classification performance, we explored the ROC curves of all models. From ROC curves we observed 0.5 was the best threshold (to obtain the highest accuracy) for most of the models. Thus, we selected a classification threshold of 0.5 (i.e., if the new location obtained a predicted probability of being flooded > 0.5, it was classified as flooded; otherwise, as non-flooded) to calculate subsequent model performance metrics. We decided to use a unified threshold of 0.5 for all validations to enhance comparison and interpretation of the results. From the classified predictions, we calculated the confusion matrix including sensitivity, specificity, and accuracy metrics (for definitions of these common terms^67,68^.

Methods References

51. Pike, D. A. Climate influences the global distribution of sea turtle nesting. *Glob. Ecol. Biogeogr.* **22**, 555–566 (2013).
52. Ehrhart, L. et al. Long-term trends in loggerhead (*Caretta caretta*) nesting and reproductive success at an important western Atlantic rookery. *Chelonian Conserv. Biol.* **13**, 173–181 (2014).
53. Patiño-Martinez, J. et al.. Globally significant nesting of the leatherback turtle (*Dermochelys coriacea*) on the Caribbean coast of Colombia and Panama. *Biol. Conserv.* **141**, 1982–1988 (2008).
54. Tadono, T. et al. Precise global DEM generation by ALOS PRISM. ISPRS *Annals of the Photogrammetry, Remote Sensing and Spatial Information Sciences,* **2**(4), 71 (2014). URL https://developers.google.com/earth-engine/datasets.
55. Lehner, B et al. New global hydrography derived from spaceborne elevation data. *Eos, Transactions, AGU*, **89**(10): 93-94 (2008).
56. Farr, T.G. et al. The shuttle radar topography mission. *Reviews of Geophysics*, **45**(2), RG2004 (2007). URL https://doi.org/10.1029/2005RG000183.
57. Jarvis, A. et al. Hole-filled SRTM for the globe Version 4. *CGIAR-CSI SRTM 90m Database,* **15**, 25-54 (2008). URL http://srtm.csi. cgiar.org.
58. Tachikawa, T et al. Characteristics of ASTER GDEM version 2. *Proceeding of 2011 IEEE International Geoscience and Remote Sensing Symposium (IGARSS)*, 3657–3660. (2011). doi:10.1109/IGARSS.2011.6050017.
59. U.S. Geological Survey, Department of the Interior, USA. 1 meter Digital Elevation Models (DEMs) - USGS National Map 3DEP Downloadable Data Collection (2020).
60. Guan, L. et al. Fusion of public DEMs based on sparse representation and adaptive regularization variation model. *ISPRS J. Photogramm. Remote Sens.* **169**, 125–134 (2020).
61. Pierce, D. ncdf4: Interface to Unidata netCDF (version 4 or earlier) format data files. R package, URL http://CRAN. R-project. org/package=ncdf4. (2012).
62. Hijmans, R. J. et al. Package ‘raster’. R package, 734 . (2015).
63. Bivand, R. et al. Package ‘rgdal’. Bindings for the Geospatial Data Abstraction Library, URL https://cran. r-project. org/web/packages/rgdal/index . (2015).
64. Woodroffe, C. D. Reef-island topography and the vulnerability of atolls to sea-level rise. *Glob. Planet. Change* **62**, 77–96 (2008).
65. Nakazawa, M. Nakazawa, M. M. Package ‘fmsb’. URL https://cran. r-proje ct. org/web/packa ges/fmsb/fmsb.pdf (2019).
66. Kopp, R. E. et al. Probabilistic 21st and 22nd century sea-level projections at a global network of tide-gauge sites. *Earth's Future*, 2, 383–406 (2014).
67. Dwivedi, Ashok Kumar. "Performance evaluation of different machine learning techniques for prediction of heart disease." *Neural Computing and Applications* 29.10 (2018): 685-693.
68. Gupta, Monika, and Anil Kumar Madan. "Diverse models for the prediction of HIV integrase inhibitory activity of substituted quinolone carboxylic acids." *Archiv der Pharmazie* 345.12 (2012): 989-1000.
69. Burnham, K. P. & Anderson, D. R. Multimodel inference: understanding AIC and BIC in model selection. *Sociol. Methods Res.* **33**, 261–304 (2004).
70. R Core Team 2020
71. Johnson, J. B., & Omland, K. S. Model selection in ecology and evolution. *Trends in Ecology & Evolution*, *19*(2), 101-108 (2004).
72. Tuszynski, J. caTools: Miscellaneous tools; I/O, moving window statistics. R package version 1.6. (2012).
73. Bates, D., Sarkar, D., Bates, M. D. & Matrix, L. The lme4 package. *R package version*, **2**, 74 (2007).
74. Barton, K. Multi-model inference (MuMIn). R package version, **1** (2016).

**Extended Data**

**Extended Data Table 1. Summary of sample size of nests.** Data from five turtle species (quantity, period, rookery).

| Species | St George beaches | Guanacabibes | Saona Island | Costa Rica beaches | St Eustatius beaches | Raine Island | Ecuador beaches |
| --- | --- | --- | --- | --- | --- | --- | --- |
| Loggerhead | 2017-19 (135) | 2007  (5) |  |  |  |  |  |
| Green |  | 2007  (158) | 2006-16  (82) |  | 2003-2014 (136) | 2019 (105) | 2013-2020 (87) |
| Hawksbill |  |  | 2006-16 (801) |  | 2003-2014 (39) |  | 2013-2020 (109) |
| Leatherback |  |  | 2006-16  (28) | 2013-15 2019-20 (1107) | 2003-2014 (39) |  |  |
| Olive ridley |  |  |  |  |  |  | 2013-2020 (11) |

**Extended Data Figure 1.**	Study site locations in the Caribbean Sea, Gulf of Mexico and Pacific Ocean. In the Caribbean and Gulf of Mexico: Mondonguillo beach, Costa Rica; Guanahacabibes peninsula, Cuba; Saona Island, Dominican Republic; Zeelandia, Turtle, Kay bay, Tumbledowndick, Crooks and Oranjebaai beaches, St Eustatius; and St George Island, Florida, USA. In the Pacific Ocean: Coast of Ecuador; and Raine Island, Australia. Map data: Google Earth.

**Extended Data Figure 2.** Sea level rise projections by the IPCC for the two Representative Concentration Pathways (RCP) for which time series projections are available: the intermediate scenario (RCP 4.5, red) and the high emissions scenario (RCP 8.5, blue) for mean log elevation (y axis) estimated by each DEM of the seven studies areas.

For

Review

**Extended Data Figure 3. Comparison of the relationship between slope (radians) and elevation (m) using empirical data (in situ measurements) and Digital Elevation Models for turtle nesting beaches in Ecuador.**

**Extended Data Figure 4.** Validation of the predictions of the models for the five Digital Elevation Models (DEMs) in comparison with the prediction of empirical data for slope and distance from sea for the rookeries in Costa Rica and Ecuador.

**Extended Data Figure 5.** CoastalDEM map of the study area at Raine Island in Australia for 2050 under the moderate source of SLR projections. The red area is predicted to be flooded. Georeferenced nest locations are plotted on the CoastalDEM map as dots. Map data: Google Earth Engine and QGIS software 3.10.0.

**Extended Data Figure 6**. Map of part of the study area of Ecuador, where it is possible to see how the CoastalDEM fails to provide the shape of the coast and the nest locations appear in the sea. Map data: Google Earth Engine and QGIS software 3.10.0.
